# Fast and furious: mapping epithelial cellular turnover into intestinal transcriptomic atlases

**DOI:** 10.1038/s44320-025-00156-8

**Published:** 2025-10-02

**Authors:** Christoph Kilian, Lorenz Adlung

**Affiliations:** 1 First Department of Medicine, University Medical Center Hamburg-Eppendorf (UKE), Hamburg, Germany; 2 Hamburg Center for Translational Immunology (HCTI), and Center for Biomedical AI (bAIome), UKE, Hamburg, Germany; 3 Mildred Scheel Cancer Career Center HaTriCS4, UKE, Hamburg, Germany

**Keywords:** Chromatin, Transcription & Genomics

## Abstract

C. Kilian & L. Adlung discuss the study by Barkai et al, in this issue of *Molecular Systems Biology*, that integrates static transcriptomic snapshots of epithelial cells by a ‘turnover score’ based on an expression calculation from resident tissue and shed cells to offer a dynamic view of cellular turnover in this tissue.

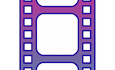

## Teaser

Understanding temporal dynamics in tissue biology is key to linking transcriptomic snapshots to physiological function. Barkai *et al*. propose a turnover score derived from gene expression of shed epithelial cells, transforming static maps into dynamic representations of cell renewal across the intestinal tract.

In single-cell biology, atlases provide detailed pictures of the cellular composition and molecular makeup of our organs. Yet, these atlases are often fundamentally static, capturing only a snapshot, i.e., a single moment in time. In reality, living tissues behave like dynamic movies with continuous cellular birth, migration, maturation, and death. Watching such a movie can feel like watching a rough stop-motion animation, with time often being approximated by statistical methods to create a smooth transition through the different cell states. Various computational modeling approaches aim to find more precise approximations of temporal transitions between cellular and molecular events. These modeling approaches are motivated by real biological processes. For example, the precision of statistically inferred pseudotime trajectories has been improved by taking mRNA splicing into consideration (Bergen et al, 2021). Moving from pseudotime to real time is also clinically relevant when considering the transition from physiological to pathological tissue conditions.

In this issue of Molecular Systems Biology, Barkai et al. address the challenge of real time in single-cell biology by proposing a gene expression-based score (Barkai et al, 2025). This score provides a longitudinal view of intestinal cellular turnover, adding a novel layer to the quantification of temporal dynamics in mucosal organs. By analyzing the transcriptomes of cells naturally shed from the intestinal epithelium, their approach transforms static spatial maps into dynamic visualizations, revealing the tempo of tissue renewal in the dramatic movie of cell birth, migration, maturation, and death.

The conceptual idea behind their approach is to exploit shed cells as a source of temporal information representing the endpoint of an epithelial cell’s life. Building on their previous work (Bahar Halpern et al, 2023), the authors first demonstrated their computational method as a proof of concept in the steady-state murine intestine. The method is simple yet powerful. They performed a linear regression of the gene expression profiles of cells, shed into the intestinal lumen (collected in intestinal washes), to those still firmly residing in the tissue. The authors extracted the residuals of the linear regression and took these as a vector to calculate a weighted sum of the gene expression. They refer to this metric as the “turnover score” in standard single-cell or spatial transcriptomics datasets.

**Fig. 1:**
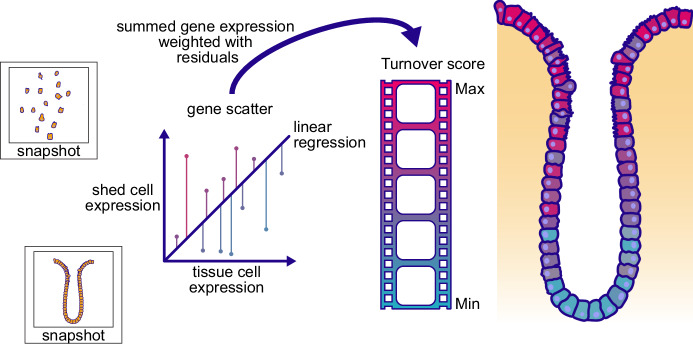
Concept behind the calculation of the turnover score to transform static snapshot information into transcriptomics atlases with temporal dynamics.

When analysed with the turnover score, such data recapitulated the well-known “conveyor belt” of cell development from stem cells in the crypt to mature enterocytes at the villus tip in the epithelium of the murine small intestine. The turnover score was indeed lowest at the bottom and peaked at the very top of the villus, precisely where most cells are shed (Fig. 1). Barkai et al. demonstrate that shed cells remain viable and show gene expression patterns similar to those of non-apoptotic cells, proving that the method accurately captures cell turnover. But to what extent does the turnover score represent biological processes? When we modeled the turnover of colonic epithelial cells deterministically during murine dextran sulfate sodium (DSS) colitis using longitudinal single-cell transcriptomics data, we estimated a kinetic turnover rate (Kilian et al, 2024). This rate can now be informed by the turnover score derived from Barkai et al. The cellular lifespan can also be approximated using the turnover score, for example, in multiscale models of the murine intestinal crypt (Gall et al, 2023). Importantly, all the data and Python code from Barkai et al. are freely available at 10.5281/zenodo.15706697.

Ultimately, the authors translated their findings from the murine to the human gastrointestinal tract. Using cells collected non-invasively from gastric fluids and fecal washes, Barkai et al. generated dynamic turnover maps of the human esophagus, stomach, duodenum, and colon. In every organ, the turnover score revealed cellular journeys. In the stomach, for example, long-lived chief cells nestled at the base of glands had low scores, while the transient, mature pit cells at the surface had high scores, reflecting their respective lifespans of months versus days. This adds long-awaited quantitative metrics and transforms static stop-motion pictures into dynamic cellular maps.

One limitation of this approach is that it relies on bulk information of shed cells. It is unclear how well such information generalizes across biological conditions in which shed cells could exhibit substantial heterogeneity. Such variability could also introduce a sampling bias depending on the location of the captured shed cells. Future studies will reveal the extent to which such covariates can be regressed out. Nevertheless, we believe that the turnover score will further advance our understanding of intestinal physiology, and we foresee that the approach will soon be applied to disease scenarios. In clinical contexts, for example, the turnover score could inform liquid biopsies from minimally invasive intestinal washes. Furthermore, the strategy of using cells at the end of their life as a timekeeper could be extended beyond the gut to other systems, such as the urogenital, respiratory, and biliary tracts. Finally, as the authors discuss, using cell-free RNA could provide information on the turnover dynamics of other internal organs where liquids or shed cells are not easily accessible. In any case, fast and furious mapping of cellular turnover in single-cell atlases is on screen, and we are eager to watch it.
